# Post-traumatic giant thrombosis of inferior vena cava induced right-sided blunt traumatic diaphragmatic injury: a case report

**DOI:** 10.1186/s40792-023-01623-w

**Published:** 2023-03-24

**Authors:** Shota Maezawa, Ryota Seo, Naotaka Motoyoshi, Takashi Irinoda

**Affiliations:** 1 Department of Emergency and Critical Care Medicine/Emergency Center, Osaki Citizen Hospital, 3-8-1 Furukawahonami, Osaki, Miyagi 989-6183 Japan; 2 Department of Cardiovascular Surgery, Osaki Citizen Hospital, 3-8-1 Furukawahonami, Osaki, Miyagi 989-6183 Japan

**Keywords:** Inferior vena cava thrombosis, Diaphragmatic injury, Blunt trauma, Pulmonary thromboembolism

## Abstract

**Background:**

Inferior vena cava thrombosis is a severe disease as it carries a higher risk of developing pulmonary embolism associated with a high mortality rate. The incidence of inferior vena cava thrombosis is extremely low and is commonly associated with outflow obstruction of the inferior vena cava. The frequency of traumatic diaphragmatic injuries is less than 1% of all traumatic injuries. In addition, it was not a typical cause of inferior vena cava obstruction. We report the case of the patient who presented with giant thrombosis of the inferior vena cava, which required surgical treatment-induced right-sided blunt traumatic diaphragmatic injury.

**Case presentation:**

A 60-year-old male presented to the emergency department with pelvic and lower leg pain. He was working on a dump truck with the bed raised position. Suddenly, the bed came down, and his body was crushed and injured. Primary CT showed a right lung contusion and elevation of the right diaphragm but no apparent liver injury. The right pleural effusion gradually worsened after admission, as the traumatic diaphragmatic injury was highly suspected. Repeat CT showed aggravation of elevation of the right-sided diaphragm, narrowing of the inferior hepatic vena cava due to left cephalic deviation of the liver, and formation of a giant thrombus in the inferior vena cava. No adverse hemodynamic effects were observed due to thrombus formation, and we performed thrombolytic therapy. The day after starting thrombolytic therapy, the patient developed pulmonary embolism due to a dropped in SpO_2_ needed oxygen, and dyspnea triggered by coughing. Thrombolytic therapy was continued after the diagnosis of pulmonary embolism. However, thrombolytic therapy was ineffective, so we decided on surgical thrombectomy and inferior vena cava filter placement. The postoperative course was not eventful, and an anticoagulant was started. The patient was transferred to the hospital on the 62nd day for rehabilitation.

**Conclusions:**

When a diaphragmatic hernia is suspected of causing hepatic hernia and narrowing of the inferior vena cava, it may be necessary to consider emergency surgical treatment to prevent secondary inferior vena cava thrombosis and fatal pulmonary embolism.

## Background

Inferior vena cava thrombosis (IVCT) is a severe disease as it carries a higher risk of developing pulmonary embolism (PE) associated with a high mortality rate. However, the incidence of IVCT is extremely low and is commonly associated with outflow obstruction of the inferior vena cava (IVC), such as Budd–Chiari Syndrome, IVC anomalies, or external compression by a mass or hematoma [1]. As IVCT can be clinically silent and may become only revealed after sudden and fatal PE, the annual incidence of IVCT is difficult to determine [2].

The frequency of traumatic diaphragmatic injuries is less than 1% of all traumatic injuries [3]. Among then, blunt traumatic diaphragmatic injury is infrequent among blunt traumatic injuries, and its frequency is reported to be less than 0.1% of all blunt traumatic patients [4]. Motor vehicle collisions are responsible for up to 90% of blunt traumatic diaphragmatic injury, with the remainder due to falls or crush injury, both of which can sufficiently elevate intra-abdominal pressure to rupture the diaphragm [3, 5]. The left-sided diaphragmatic injury accounts for more than 70% of injuries, and right-sided diaphragmatic injuries are more likely to be complicated by liver injuries [6]. Diagnosis of traumatic diaphragmatic injury can be made with plain chest X-ray and computed tomography (CT) but is not easy without intra-thoracic deviation of abdominal organs. In hemodynamically stable patients who are non-operatively managed, the rate of initially missed diaphragmatic injury is between 12% and 66% [7, 8]. In a series of 57 patients with blunt traumatic diaphragmatic injury, diagnosis of diaphragmatic injury was not made until laparotomy was performed for associated injuries in 42% of patients [8].

Here we present the case of a 60-year-old male with a giant IVCT due to right-sided diaphragmatic injury and hernia, which was diagnosed 9 days after trauma and required surgical treatment.

## Case presentation

A 60-year-old Japanese man presented to the emergency department (ED) with pelvic and lower leg pain. He was working on a dump truck with the bed raised position for inspection. Unfortunately, the bed came down suddenly, and his body was crushed and injured. When he showed up ED, he was hemodynamically stable, and there was no sign of shock except tachypnea and tachycardia.

**Fig. 1:**
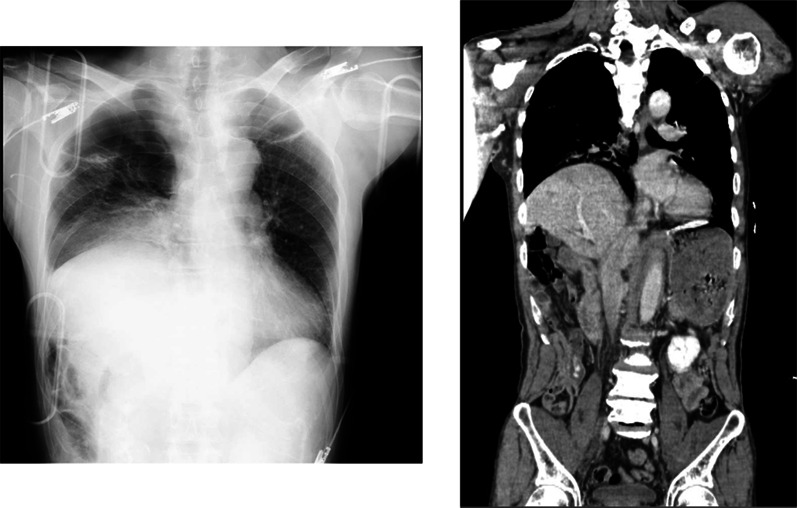
Chest X-ray and CT scan findings on the day of admission. The enhanced CT scan and chest X-ray shows right diaphragm elevation and pulmonary contusion

Primary CT and chest X-ray showed a right lung contusion and elevation of the right diaphragm but no apparent liver injury (Fig. 1). The pelvic and lower extremities fractures were observed. At this point, the right-sided diaphragmatic injury could not be ruled out. However, the patient was under observation because of the hemodynamically stable and had no thoracoabdominal injuries requiring emergency surgery.

The right pleural effusion gradually worsened after admission, and a chest tube was inserted on the fourth day of admission, which showed drainage of bloody pleural effusion. Pleural effusion from the chest tube continued to more than 300 mL daily. Therefore, the contrast CT scan was re-performed on the ninth day of admission, as the traumatic diaphragmatic injury was suspected.

**Fig. 2:**
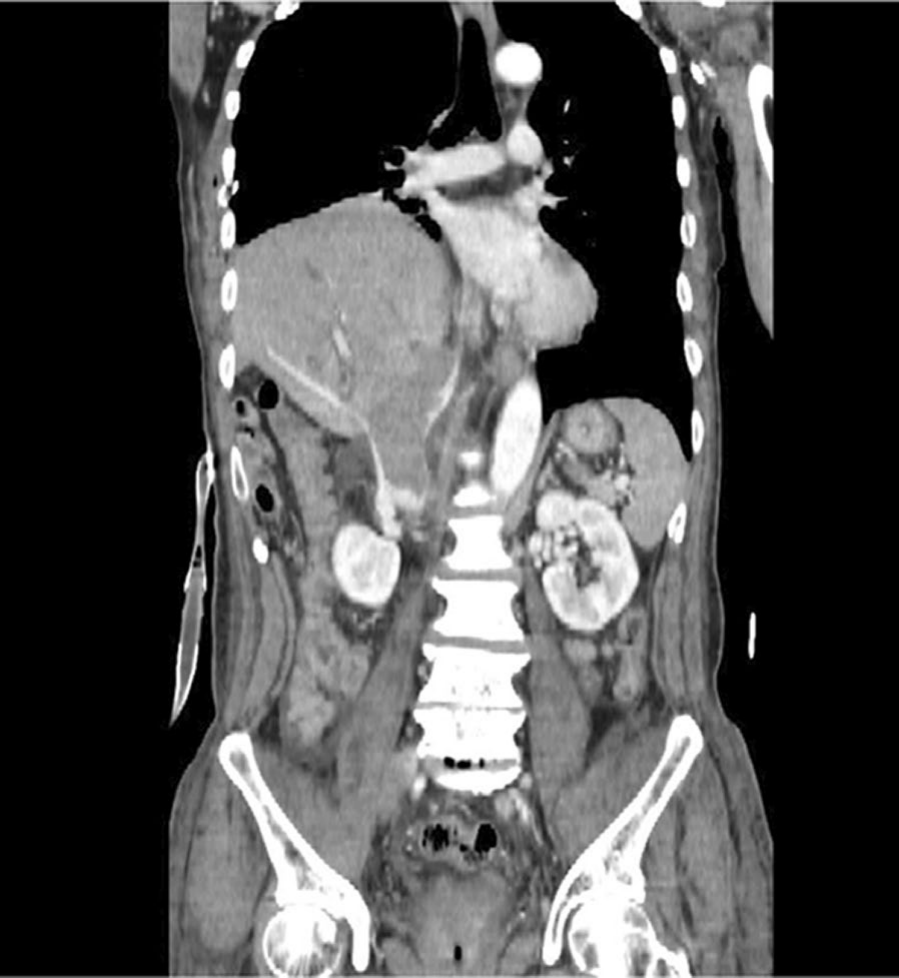
Follow-up enhanced CT scan findings on the ninth day of admission. Compared to the CT scan on the admission, the liver was more elevated, and a diagnosis of traumatic diaphragmatic injury was made. The diaphragmatic hernia caused severe narrowing of the inferior hepatic vena cava and formed a 13 cm × 3 cm giant thrombus in the inferior vena cava

Repeat CT showed aggravation of elevation of the right-sided diaphragm, narrowing of the inferior hepatic vena cava due to left cephalic deviation of the liver, and formation of a 13 cm × 3 cm thrombus in the IVC (Fig. 2). There was no apparent worsening of bilateral leg edema or physical findings in the abdomen. No adverse hemodynamic effects were observed due to thrombus formation, and we performed thrombolytic therapy with urokinase and heparin. The dose of urokinase was halved every 2 days, starting at 4400 IU/kg, and continued for 6 days, and unfractionated heparin 20,000 units per day.

**Fig. 3:**
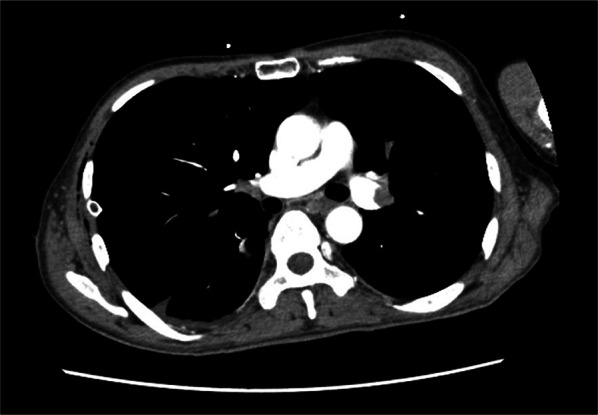
Enhanced CT scan findings after sudden dyspnea and dropped SpO_2_. The enhanced CT scan showed Pulmonary thromboembolism due to thrombus in the left main pulmonary artery

The day after starting thrombolytic therapy, the patient developed pulmonary thromboembolism due to a dropped in SpO_2_ needed oxygen, and dyspnea triggered by coughing (Fig. 3). Fortunately, the patient’s hemodynamic status was not adversely affected, and the decision was made to continue the current thrombolytic therapy.

However, a follow-up ultrasound on the day urokinase administration was terminated showed residual thrombus in the IVC, and there was no apparent change in the thrombus size before or after the initiation of anticoagulation. The patient was considered ineligible for further thrombolytic therapy and prepared for surgical thrombectomy because of the high risk of developing a fatal PE.

Open-heart surgical thrombectomy using cardiopulmonary bypass with a median sternotomy and an IVC filter placement was performed on the 26th day of admission. A Hybrid Operating room was used, because fluoroscopic equipment was needed during surgery.

Through a median sternotomy, the pericardium was incised and taped to the ascending aorta and superior vena cava. Cardiopulmonary bypass was established with cannulation of the ascending aorta, the superior vena cava, and the right atrium. The pericardium around the IVC was lifted, and the IVC was exposed. Then, opened the right atrium and blood flow from IVC was confirmed. An introducer sheath was inserted into the right external iliac vein, and a guide wire was advanced and caught in the right atrium. Intravascular ultrasound confirmed a large amount of thrombus in the IVC from the renal vein junction to the hepatic vein junction. At the beginning of the thrombectomy, an intravenous occlusion balloon was advanced from the right atrium to the IVC. However, the balloon could not be advanced to the proper position due to the flexure of the IVC. Therefore, a balloon was pushed up from the external iliac vein to guide the thrombus into the right atrium, and 15 g of fresh thrombus was removed. Thrombectomy for pulmonary artery was not performed, because it did not affect hemodynamics and did not require surgical thrombectomy.

The postoperative course was not eventful, and an anticoagulant therapy with direct oral anticoagulant (DOAC) was started on post operative day 6. The patient was on bed rest for a while due to the pelvic fracture, but the bed rest level was gradually increased, and rehabilitation treatment was continued.

After orthopedic surgery for a right ankle fracture, the patient was transferred to the hospital on the 62nd day for rehabilitation. All tests performed to evaluate congenital thrombophilia were negative.

## Discussion

This case led to the diagnosis of right-sided traumatic diaphragmatic injury and diaphragmatic hernia approximately 10 days after the injury and the formation of a giant thrombus in the IVC, which required surgical thrombectomy.

Inferior vena cava thrombosis is a rare event and is associated with known risk factors for venous thromboembolism. Pulmonary embolism can occur in roughly 25% of patients presenting with IVCT [9]. In the previous report, there were 10 cases of traumatic inferior vena cava thrombosis requiring surgery (Table 1) [10–19], and there were no reports of diaphragmatic hernia as a cause of thrombus formation as in this case. Since 2000, postoperative anticoagulation has been administered in 80% of cases. The time from traumatic injury to the onset of thrombosis were varied from the day of injury to 4 years, suggesting that the triggering mechanism of IVCT may differ. The pathogenesis of IVCT associated with abdominal trauma generally occurs in the following manner. 1, Hepatic vein thrombosis, which extends into the IVC; 2, Endothelial injury of the IVC wall; 3, IVC flow stasis caused by the compression with an around IVC or a retroperitoneal hematoma; 4, Hypercoagulable and hypofibrinolytic state after major trauma [17].

**Table 1:** Previous reports requiring surgical treatment for traumatic secondary IVCT

Author, year	Complicated injuries	Days from injury to onset	Treatment
Grmoljez et al. [10] 1976	IVC laceration	6 weeks	Thrombectomy
Mayzlik et al. [11] 1992	Rib fracture	2 months	Thrombectomy
Kimoto et al. [12] 1998	Hepatic injury	35 days	Thrombectomy
Balian et al. [13] 1998	Hepatic injury Renal injury	3 days	Thrombectomy, Anticoagulation
Cellarier et al. [14] 1999	Blunt abdominal trauma	3 months	Thrombectomy, Anticoagulation
Fujii et al. [15] 2002	Hepatic injury	30 days	Thrombectomy, Atriotomy, Anticoagulation
Ushijima et al. [16] 2007	Pancreatic injury	4 years	Thrombectomy, Atriotomy, Anticoagulation
Hamamoto et al. [17] 2013	Hepatic injury	1 month	Thrombectomy, Anticoagulation
Sabzi et al. [18] 2016	Blunt abdominal trauma	10 days	Atriotomy, Pulmonary embolectomy, Anticoagulation
Salloum et al. [19] 2016	Hepatic injury	On admission	Thrombectomy
Our case	Right-sided diaphragmatic injury	9 days	Thrombectomy, Anticoagulation

Anticoagulation is the standard treatment for IVCT [1], and early initiation of anticoagulation is recommended. However, the combination of bleeding from other organ injuries limits early initiation after trauma. In addition, systemic thrombolytic therapy has a higher rate of complete clot lysis than anticoagulation alone for proximal DVT, and it is not highly recommended for active use due to the high frequency of major bleeding complications [20]. In the case of secondary IVCT, including IVC stenosis, such as in the present case, the fix of the stenosis was considered. However, a large IVCT had already formed, and thrombolytic therapy was ineffective. Therefore, surgical thrombectomy and IVC filter placement had priority, because the patient was at high risk of developing fatal PE in this case. The primary surgical repair for diaphragmatic hernia was abandoned because of the high risk of bleeding due to the need for full heparinization by open heart surgery using cardiopulmonary bypass.

The mechanism of blunt traumatic diaphragmatic injury is increased intra-abdominal pressure. The remainder is due to falls or crush injury, which can sufficiently elevate intra-abdominal pressure to rupture the diaphragm [6, 21].

Diagnosis of traumatic diaphragmatic injury is often difficult by clinical images such as CT and X-ray during a traumatic survey [7]. Guth et al. reported 42% of diaphragmatic injuries were diagnosed as other organ injuries during laparotomy [8].

For right-sided traumatic diaphragmatic injuries, basically, surgical treatment is recommended. Although, the Eastern Association for the Surgery of Trauma (EAST) guidelines suggest initial nonoperative management rather than operative repair in hemodynamically stable patients without signs of peritonitis [21]. However, early surgical treatment should be considered for right-sided diaphragmatic injuries with a large hepatic hernia because of the possibility of IVCT and PE due to IVC compression, as in this case.

As a surgical technique for diaphragmatic injury, other abdominal organ injuries are involved; laparotomy is often chosen. In contrast, open thoracotomy or thoracoscopic surgery is also an option for patients without abdominal organ injuries. In the acute phase of injury, direct suture using non-absorbable threads is considered, but if the diaphragmatic defect is significant or has been days since the injury, patch closure using an artificial material is also an option [22].

The diagnosis and treatment of diaphragmatic injuries with stable vital signs and no evidence of peritonitis can be challenging. In the current trauma practice, where nonoperative treatment is increasingly the treatment of choice, emergency laparoscopy or thoracoscopic surgery should be considered for diagnosis and treatment even when vital signs are stable if a diaphragmatic injury cannot be ruled out.

## Conclusion

A large right-sided diaphragmatic hernia can cause secondary IVCT and lead to the development of a potentially fatal PE. Therefore, when a diaphragmatic hernia is suspected of causing hepatic hernia and narrowing of the IVC, it may be necessary to consider emergency surgical treatment to prevent fatal thrombosis.

## Data Availability

All data generated or analyzed during this study are included in this manuscript.

## Abbreviations

IVCT: Inferior vena cava thrombosis
PE: Pulmonary embolism
IVC: Inferior vena cava
CT: Computed tomography
ED: Emergency department
DOAC: Direct oral anticoagulant
